# Prevalence of mental illness in patients with obstructive sleep apnea – A cross-sectional study from Kashmir, India

**DOI:** 10.1016/j.amsu.2022.104056

**Published:** 2022-07-06

**Authors:** Sheikh Shoib, Irfan Ullah, Sachin Nagendrappa, Anab Rehan Taseer, Domenico De Berardis, Manjeet Singh, Muhammad Sohaib Asghar

**Affiliations:** a Department of Psychiatry, Jawahar Lal Nehru Memorial Hospital (JLNMH), Rainawari, Srinagar, Jammu and Kashmir, 190003, India; b Kabir Medical College, Gandhara University, Peshawar, Pakistan; c Department of Psychiatry, National Institute of Mental Health and Neurosciences (NIMHANS), Bengaluru, 560029, India; d Internal Medicine, Hayatabad Medical Complex, Peshawar, Pakistan; e NHS, Department of Mental Health, Psychiatric Service for Diagnosis and Treatment, Hospital “G. Mazzini”, ASL 4, 64100, Teramo, Italy; f Liaquat National Hospital and Medical College, Karachi, Pakistan; g Dow University of Health Sciences-Ojha Campus, Karachi, Pakistan

**Keywords:** Mental disorders, Obstructive sleep apnea, Psychiatric disorders, Screening, Sleep problem

## Abstract

**Aim:**

The study aimed to evaluate the prevalence of mental illness in obstructive sleep apnea (OSA) and to examine whether patients with obstructive sleep apnea require screening for mental illness.

**Methods:**

We performed polysomnography studies of patients that were referred from various subspecialty clinics in Kashmir from Jan 2020–December 2020. using the Mini-International Neuropsychiatric Interview (MINI plus) scale to make a psychiatric diagnosis. We administered the General Health Questionnaire – 28 (GHQ – 28), Hamilton Depression Rating Scale (HAM-D), and Hamilton Anxiety Rating Scale (HAM-A) to patients. Descriptive statistics and correlations were used for data analysis.

**Results:**

182 patients underwent polysomnography, 85 (46.7%) of which were suffering from mental illness Based on the Apnea-Hypopnea Index score, 8 (4.39%) patients had mild, 35 (41.1%) had moderate and 42 (49.4%) had severe OSA. The mean GHQ -28 score was significantly higher in patients with Obstructive sleep apnea (p < 0.001) (11.34 ± 8.2) as compared to non-Obstructive sleep apnea patients (1.98 ± 4.38).

**Conclusions:**

This study demonstrates the increased prevalence of anxiety and depression in patients with OSA. Therefore, we recommend timely screening for any mental health issues in patients with OSA and necessary interventions to address the issues, thus preventing mental health morbidities in patients with OSA this would help subsequently in an improved lifestyle.

## Highlights

• This study highlighted the prevalence of mental illness in obstructive sleep apnea (OSA).
• Almost one-half of the patients diagnosed with OSA had mental illness.
• Appropriate measures should be undertaken to control prevalence of anxiety and depression in patients with OSA.

## 1 Introduction

Obstructive Sleep Apnea (OSA) is one of the respiratory sleep-related disorders, in which patients suffer from hindered breathing during sleep. The recurring upper airway obstruction causes repeated episodes of decreased or no airflow during sleep. This results in frequent arousals during sleep, leading to fragmented sleep [1]. Besides, OSA includes other symptoms such as daytime drowsiness, tiredness, irritability, forgetfulness, and a diminished quality of life [2]. OSA is a very common sleep disorder, The apnea-hypopnea index (AHI) is a scale is used the sleep apnea severity (index less than 5 is considered normal. For an Apnea-Hypopnea Index from 5 to 15 denotes mild sleep apnea. Fifteen to 30 is moderate, while a greater than 30 is considered severe) and global prevalence of OSA in the general population is estimated to be 9–38% (AHI ≥5) and 6%–17% (AHI ≥15) [3]. The prevalence of OSA in Asian countries, in general, varies from 3.7% to 97.3%, whereas in India (where the present research was carried out), the rate is reported to be 13.7% [4,5]. Studies have shown OSA cases are unrecognized and untreated in health care settings across the world, because of a lack of an interdisciplinary approach for treatment and management of OSA [6]. For example, more than 80% of individuals with moderate to severe OSA are not clinically diagnosed in the US population. We can expect this more severe in a developing country like India [7]. OSA may affect multiple organs resulting in different systemic disorders; for instance – cardiovascular diseases such as stroke, hypertension, coronary artery disease, and metabolic disorders such as diabetes, dyslipidemia, hypertension are highly associated with OSA [8]. In addition, it has been also shown that OSA affects multiple organs due to sympathetic stimulation caused by repeated arousal during sleep [9]. In addition to the physical complications, multiple psychiatric disorders are also commonly reported among persons with OSA [10]. These mental disorders may be provoked by the biological and/or psychosocial consequences of OSA [11]. Even though psychiatric disorders are commonly reported in people with OSA; the association between the two is not well established in India [[12], [13], [14]]. Many mental disorders can mimic symptoms of OSA and can cause long-term negative consequences such as anxiety and depression and result in the development of cognitive impairment [15]. In India, the evidence considering mental health comorbidities in persons with OSA is scarce [16,17]. The mental health issues in OSA are underestimated and unrecognized in the whole of India, particularly in Jammu and Kashmir state. This study aims to examine OSA and its correlation with psychiatric disorders in the Jammu and Kashmir state. We aim to discuss the results concerning the existence of knowledge gaps and facilitating effective policy making.

## 2 Methods

### 2.1 Participants and study site

This cross-sectional study was carried out at the Modern Hospital Srinagar. The particular hospital was selected for including patients as Standard Polysomnography laboratory facilities are only available in the tertiary care hospital and OSA patients across Jammu and Kashmir state, India, is usually referred to the hospital. Of the available OSA patients, the study excluded as of – (i) being patients of nocturnal oxygen supplementation, (ii) having unstable cardiopulmonary, neurological, or psychiatric diseases, (iii) having upper airway surgery, and (iv) using positive airway pressure therapy or oral appliances.

## 3 Measurements

### 3.1 Polysomnography

Demographic data, general medical history, clinical information from and sleep-related complaints at the initial visit as well as polysomnography results were recorded. Anthropometric measurements including height, weight, and neck circumference were measured in all patients and the blood pressure of each participant was recorded. PSG recordings were started based on the subject's usual domestic sleeping habits and each patient was recorded for a minimum of 7 h.

During 30 s in Non-REM sleep stages, 1–4 sleep, and in REM sleep recordings were scored as per guidelines [18]. Respiratory events and microarousals were scored according to conventional criteria as defined by American Sleep Disorders Association [19]. The criteria of Rechtschaffen and Kales were used to define sleep [18]. Apnea was defined as a termination of airflow for at least 10s [20]. Hypopnea was defined as a decrease in thoracic-abdominal movements of 50% or more and a decrease of the oxygen saturation of 4% or more. The Apnea Hypopnea Index (AHI) was calculated as the number of apneas and hypopneas per hour of total sleep time. Obstructive sleep apnea was defined as an AHI of 5 or more apneas/hypopneas per hour [19]. Epworth Sleepiness Scale was used to measure daytime sleepiness [21]. A score of more than 9 points was considered as excessive daytime sleepiness. We defined OSA severity categories according to commonly used clinical cutoffs: No OSA (AHI <5), Mild OSA (AHI ≥5 but <15), Moderate OSA (AHI ≥15 but <30), and severe OSA (AHI ≥30) as per American Sleep Disorders Association [19].

### 3.2 Measurement of psychopathology

General Health Questionnaire – 28 (GHQ – 28) is a widely used instrument for screening minor psychiatric morbidity [22]. The GHQ-28 is used to indicate psychological well-being and detect possible cases of psychiatric disorders [22]. The OSA patients were then assessed for psychiatric diagnosis using MINI International Neuropsychiatric Interview Schedule Plus (MINI PLUS) [23]. MINI-International Neuropsychiatric Interview (MINI-Plus) is a structured diagnostic interview, developed to assess the diagnoses of psychiatric patients according to DSM-IV and ICD-10 criteria in less time than other diagnostic interviews such as the Structured Clinical Interview for DSM-IV disorders. This was followed by the administration of the Hamilton Depression Rating Scale (HAM-D) [24] and the Hamilton Anxiety Rating Scale [25].

### 3.3 Ethics (and consent to participate)

All participants gave written informed consent before PSG. Besides, for involving in the psychopathology study, all patients were informed about the nature of the research within the hospital and gave informed consent to participate in the study. Information sheets and preliminary interviews made it clear that the choice to consent or otherwise would have no bearing on the treatment offered. The anonymity of the subjects was ensured by replacing patient names with unique identifying numbers before the statistical procedures began. The study conformed to the provisions of the Declaration of Helsinki. Formal research protocol registration for our study was obtained from the ethical review committee of the DHK-1, Srinagar, Jammu, and Kashmir, India (UIN: DHK-1/2020). STROCSS guideline checklist was followed to report the findings [26].

### 3.4 Statistical analysis

Descriptive statistics were used for measuring mean and percentage. The Kolmogorov–Smirnov test was used to test for normality of the data and the Levene test to study the variance. The variables were analyzed with the chi-square test or with Fisher's exact test if at least one cell had an expected count <5. Pearson's coefficient was used to test the correlation between quantitative variables. The level of significance was set at α = 0.05. SPSS 24.0 was used for data analyses (SPSS Inc., Chicago, IL, USA).

## 4 Results

Out of 182 patients who underwent PSG, 85 suffered from psychiatric disorders with a mean age of 58.60 years. The demographic, clinical, and PSG characteristics of the study population are given in Table 1. Based on the AHI score, 8 (4.39%) patients had mild, 35 (41.1%) had moderate and 42 (49.9%) had severe OSA (Table 2). Compared to non-psychiatric patients moderate and severe OSA was significantly higher in psychiatric patients (p < 0.005). The main presenting symptoms of patients with OSA were snoring 153 (97.5%), daytime sleepiness 140 (84.1%), disturbed nocturnal sleep 121(66.5%), nocturia 108 (59.3%), and witnessed apneas 73 (40.1%). All these symptoms were more common in patients with OSA compared to non-OSA patients (Table 3).

**Table 1:** Basic characteristics and PSG findings of the study population (N = 182).

Mean ± SD	Mean ± SD
Age (years)	54.89 ± 12.89
BMI	31.09 ± 4.61
Neck circumference (cm)	40.27 ± 3.38
AHI	21.79 ± 12.04
ESS	14.93 ± 3.93
Sleep efficiency	72.22 ± 9.31
Sleep latency (min)	21.19 ± 9.06
REM latency (min)	74.62 ± 14.32
Awake SpO2	93.16 ± 4.02
Nocturnal SpO2	86.01 ± 6.62
ODI –	21.68 ± 14.97

BMI- Body mass index.

ODI –(Overnight desaturation index).

AHI- Apnea hypopnea index.

ESS- Epsworth sleepness scale.

**Table 2:** Severity of OSA based on AHI in different psychiatric disorders.

	All patients	Total, Psychiatric disorders	Depression	Anxiety disorders	GAD	OCD	Panic disorder	Miscellaneous(social phobia)
Mild	27(17. %)	8 (4.39%)	3 (6.8%)	3 (15.0%)	1 (25.0%)	0 (0.0%)	1 (7.14%)	0 (0%)
Moderate	69(43.9%)	35 (41.1%)	18 (40.9%)	8 (40.0%)	1 (25.0%)	0 (0.0%)	7 (50%)	1 (50%)
Severe	61(38.9%)	42 (49.4%)	23 (52.3%)	9 (45.0%)	2 (50.0%)	1 (100.0%)	6 (42.8%)	1 (50%)
p-value	–	p < 0.005	0.034	0.832	–	–	–	–

**Table 3:** Patient Presentation in psychiatric morbidity - with and without psychiatric morbidity.

		No psychiatric morbidity	Psychiatric morbidity	Total	p-value
Snoring	N	20	153	173	<0.001
	%	80.0%	97.5%	95.1%	
Witnessed Apneas	N	0	73	73	<0.001
	%	0.0%	46.5%	40.1%	
Disturbed sleep	N	10	111	121	<0.001
	%	40.0%	70.7%	66.5%	
Daytime Sleepiness	N	13	140	153	<0.001
	%	52.0%	89.2%	84.1%	
Nocturia	N	7	101	108	<0.001
	%	28.0%	64.3%	59.3%	

The distribution of various psychiatric disorders is given in Table 4 with total psychiatric morbidity of 46.7% (85 subjects). Depression constitutes 25.8% (47) and anxiety disorder constitute 11.5% [21]. The mean HAM-D score was significantly higher in OSA patients (p = 0.0001) than in non-OSA patients (mean ± SD = 17.35 ± 5.45) as compared to non-depressive (mean ± SD 8.64 ± 6.24). The mean HAM-A score was not significantly higher in OSA patients (p > 0.05), (Mean ± SD = 12.1 ± 5.45) as compared to non-OSA (Mean ± SD 6.29 ± 6.24) (Table 5). The mean GHQ-28 score was significantly higher in OSA patients (p < 0.05).

**Table 4:** Distribution of psychiatric disorders in OSA.

	Frequency	Percentage
Total, Psychiatric morbidity	85	46.7
MDD	47	25.8
Anxiety disorders	21	11.5
GAD	4	2.2
OCD	1	.5
Panic disorder	14	7.7
Miscellaneous	2	1.1

**Table 5:** HAM-A and HAM-D and GHQ scores in patients with OSA and without OSA.

Variables	Total	OSA	No OSA	p-value
HAM-D (Mean ± SD)	10.85 ± 7.13	17.35 ± 5.45	8.64 ± 6.24	<0.001
HAM-A (Mean ± SD)	10.63 ± 7.13	12.1 ± 5.8	6.29 ± 6.24	>0.05
GHQ Score (Mean ± SD)	8.85 ± 7.13	11.34 ± 8.2	1.98 ± 4.38	<0.05

## 5 Discussion

Obstructive sleep apnea (OSA) is a well-known risk factor for many systemic disorders and psychiatric disorders. This is the first published study of the prevalence of psychiatric disorders in OSA from Kashmir, India. Our results show that the frequency of comorbid mental illness is significantly higher in individuals with sleep apnea. This pattern was mainly prominent for depression and less for anxiety. Total psychiatric morbidity of 46.7% was present in the studied population. All types of disorders mental illness were more common among patients with OSA; however, depression and anxiety disorders were most distinct. The mean GHQ-the score was significantly higher in OSA patients (Mean ± SD = 11.34 ± 8.2) as compared to non-OSA. Depression (25.8%) and anxiety disorder (11.5%) were the leading diagnoses. Most of the studies have shown a significant association between psychiatric disorders and OSA [16,17]. However, few studies have ruled out any association between OSA and psychiatric disorders [27,28]. The mental changes may be provoked by the biological or psychosocial consequences of OSA [10]. These mental disorders can have a harmful impact on the quality of life and their neurocognitive functioning in patients with OSA [29]. The findings of our study suggest that mental disorders are highly prevalent in OSA primary care settings. Most clinicians do not suspect this important comorbidity of OSA, in the beginning, resulting in delayed diagnosis. There needs to be a holistic approach in evaluating OSA to consider the possibility of mental health morbidities, assessment of mental illness should be incorporated in OSA management. An important observation of our study was that severe and moderate OSA were significantly higher in patients suffering from some form of mental illness compared to those not having a mental illness. In the present study, 25.8% of patients with depression had OSA indicating that OSA is a highly prevalent co-morbidity in depression supported by Beutler et al. [30] who studied the psychological characteristics of 50 patients with sleep apnea and narcolepsy in a case-controlled age matched groups. The co-morbidity between OSA and anxiety has also been observed [31]. The prevalence of anxiety disorder in patients with sleep apnea was found to be 11.5%. This finding is supported by Borak et al. [32] in an observational study. Yue et al. replicated a similar finding of higher anxiety scores in a case-controlled study of 30 patients with OSA [33]. Timely detection of psychiatric disorders will lead to adequate management [[34], [35], [36], [37]].

### 5.1 Limitation of the study

Some of the limitations of the study were a relatively modest sample size. Another limitation of our study is its cross-sectional design fails to determine causality, other factors like diabetes, hypertension, obesity might have associated depressive disorders, anxiety, etc. Furthermore, we have used standardized rating scales to make a diagnosis of mental illness.

## 6 Conclusions

A vigilant mental assessment would be an apt response to this urgent condition to assess mental health conditions in OSA and screening and timely diagnosis may improve the quality of life of the patient. Further epidemiological studies are necessary to gauge the degree of the problem and therefore, screening for Obstructive sleep apnea for mental illness would facilitate better interventions and consequently better outcomes. Future research directions should focus on studying the therapeutic options for such vulnerable patient population.

## Ethical approval

Ethical approval was taken in this study from ethical review committee of Modern Hospital Srinagar (Ref no: DHK-1/2020).

## Sources funding

None.

## Author contribution

S.S, I.U, and S.N conceived the idea; A.R.T, D.D.B, M.S, and S.S collected the data; M.S.A and I.U analyzed and interpreted the data; M.S, S.N, S.S, A.R.T, I.U, and D.D.B did write up of the manuscript; and finally, I.U, M.S.A and S.N reviewed and revised the manuscript for intellectual content critically. All authors approved the final version of the manuscript.

## Trail register number

1. Name of the registry: Modern Hospital, Srinagar, Jammu, and Kashmir, India
2. Unique Identifying number or registration ID: DHK-1/2020
3. Name of the registry: Modern Hospital, Srinagar, Jammu, and Kashmir, India

## Guarantor

Muhammad Sohaib Asghar.

## Provenance and peer review

Externally peer reviewed, not commissioned.

## Consent

Informed consent was obtained from each participant before enrolment in the study.

## Declaration of competing interest

None.

## References

[1] Malik J.A., Masoodi S.R., Shoib S. (2017). Obstructive sleep apnea in Type 2 diabetes and impact of continuous positive airway pressure therapy on glycemic control. Indian J. Endocrinol. Metabol..

[2] Seneviratne U., Puvanendran K. (2004). Excessive daytime sleepiness in obstructive sleep apnea: prevalence, severity, and predictors. Sleep Med..

[3] Senaratna C.V., Perret J.L., Lodge C.J., Lowe A.J., Campbell B.E., Matheson M.C., Dharmage S.C. (2017). Prevalence of obstructive sleep apnea in the general population: a systematic review. Sleep Med. Rev..

[4] Mirrakhimov A.E., Sooronbaev T., Mirrakhimov E.M. (2013). Prevalence of obstructive sleep apnea in Asian adults: a systematic review of the literature. BMC Pulm. Med..

[5] Reddy E.V., Kadhiravan T., Mishra H.K., Sreenivas V., Handa K.K., Sinha S., Sharma S.K. (2009). Prevalence and risk factors of obstructive sleep apnea among middle-aged urban Indians: a community-based study. Sleep Med..

[6] Lim D.C., Sutherland K., Cistulli P.A., Pack A.I. (2017). P4 medicine approach to obstructive sleep apnoea. Respirology.

[7] Kapur V., Strohl K.P., Redline S., Iber C., O’connor G., Nieto J. (2002). Underdiagnosis of sleep apnea syndrome in US communities. Sleep Breath..

[8] Franklin K.A., Lindberg E. (2015). Obstructive sleep apnea is a common disorder in the population—a review on the epidemiology of sleep apnea. J. Thorac. Dis..

[9] Ziegler M.G., Nelesen R., Mills P., Ancoli-Israel S., Kennedy B., Dimsdale J.E. (1997). Sleep apnea, norepinephrine-release rate, and daytime hypertension. Sleep.

[10] Ohayon M.M. (2003). The effects of breathing-related sleep disorders on mood disturbances in the general population. J. Clin. Psychiatr..

[11] Pochat M.C., Ferber, Lemoine P. (1993). Depressive symptomatology and sleep apnea syndrome. L'Encephale.

[12] Borak J., Cieślicki J., Szelenberger W., Wilczak-Szadowska H., Koziej M., Zieliński J. (1994).

[13] Kaplan R. (1992). Obstructive sleep apnoea and depression—diagnostic and treatment implications. Aust. N. Z. J. Psychiatr..

[14] Kalucy M.J., Grunstein R., Lambert T., Glozier N. (2013). Obstructive sleep apnoea and schizophrenia–A research agenda. Sleep Med. Rev..

[15] Monti J.M., Monti D. (2004). Sleep in schizophrenia patients and the effects of antipsychotic drugs. Sleep Med. Rev..

[16] Kant S., Kapoor N., Verma A.K., Verma S.K., Kushwaha R.A.S., Kumar S. (2019). Psychiatric manifestations in the patients of obstructive sleep apnea at tertiary care center of Northern India. Indian J. Respir. Care.

[17] Shoib S., Malik J.A., Masoodi S. (2017). Depression as a manifestation of obstructive sleep apnea. J. Neurosci. Rural Pract..

[18] Rechtschaffen A., Kales A. (1968). A Manual of Standardized Terminology, Techniques and Scoring System of Sleep Stages in Human Subjects.

[19] (1992). EEG arousals: scoring rules and examples: a preliminary report from the sleep disorders Atlas task force of the American sleep disorders association. Sleep.

[20] Stradling J.R., Davies R.J.O. (2004). Sleep· 1: obstructive sleep apnoea/hypopnoea syndrome: definitions, epidemiology, and natural history. Thorax.

[21] Johns M.W. (1992). Reliability and factor analysis of the Epworth sleepiness scale. Sleep.

[22] Goldberg D.P., Hillier V.F. (1979). A scaled version of the general health Questionnaire. Psychol. Med..

[23] Lecrubier Y., Sheehan D.V., Weiller E., Amorim P., Bonora I., Sheehan K.H. (1997). The Mini International Neuropsychiatric Interview (MINI). A short diagnostic structured interview: reliability and validity according to the CIDI. Eur. Psychiatr..

[24] Hamilton M. (1960). A rating scale for depression. J. Neurol. Neurosurg. Psychiatr..

[25] Hamilton M. (1959). Hamilton anxiety scale. Group.

[26] Mathew G., Agha R., for the STROCSS Group (2021). STROCSS 2021: strengthening the Reporting of cohort, cross-sectional and case-control studies in Surgery. Int. J. Surg..

[27] Balan S., Spivak B., Mester R., Leibovitz A., Habot B., Weizman A. (1998). Psychiatric and polysomnographic evaluation of sleep disturbances. J. Affect. Disord..

[28] Millman R.P., Fogel B.S., McNamara M.E., Carlisle C.C. (1989). Depression as a manifestation of obstructive sleep apnea: reversal with nasal continuous positive airway pressure. J. Clin. Psychiatr..

[29] Flemons W.W., Tsai W. (1997). Quality of life consequences of sleep-disordered breathing. J. Allergy Clin. Immunol..

[30] Beutler L.E., Ware J.C., Karacan I., Thornby J.I. (1981). Differentiating psychological characteristics of patients with sleep apnea and narcolepsy. Sleep.

[31] Mosko S., Zetin M., Glen S., Garber D., DeAntonio M., Sassin J. (1989). Self‐reported depressive symptomatology, mood ratings, and treatment outcome in sleep disorders patients. J. Clin. Psychol..

[32] Borak J., Cieślicki J., Koziej M., Matuszewski A., Zieliński J. (1996). Effects of CPAP treatment on psychological status in patients with severe obstructive sleep apnoea. J. Sleep Res..

[33] Yue W., Hao W., Liu P., Liu T., Ni M., Guo Q. (2003). A case—control study on psychological symptoms in sleep apnea-hypopnea syndrome. Can. J. Psychiatr..

[34] Cassel W., Henn-Kolter C., Pilot A. (1992). Personality changes in sleep apnea. J. Sleep Res..

[35] Quan S.F., Gillin J.C., Littner M.R., Shepard J.W. (1999). Sleep-related breathing disorders in adults: recommendations for syndrome definition and measurement techniques in clinical research. editorials. Sleep (New York, NY).

[36] Reynolds C.F., Kupfer D.J., McEachran A.B., Taska L.S., Sewitch D.E., Coble P.A. (1984). Depressive psychopathology in male sleep apneics. J. Clin. Psychiatr..

[37] Shoib S., Das S. (2020). Factors predicting the presence of depression in obstructive sleep apnea. Ind. Psychiatr. J..

